# A critical systematic review of extracellular vesicle clinical trials

**DOI:** 10.1002/jev2.12510

**Published:** 2024-09-27

**Authors:** Rachel R. Mizenko, Madison Feaver, Batuhan T. Bozkurt, Neona Lowe, Bryan Nguyen, Kuan‐Wei Huang, Aijun Wang, Randy P. Carney

**Affiliations:** ^1^ Department of Biomedical Engineering University of California Davis California USA; ^2^ Department of Surgery University of California Davis California USA

**Keywords:** diagnostics, drug delivery vehicles, exosomes, heterogeneity, therapeutics

## Abstract

This systematic review examines the landscape of extracellular vesicle (EV)‐related clinical trials to elucidate the field's trends in clinical applications and EV‐related methodologies, with an additional focus on the acknowledgement of EV subpopulations. By analysing data from public reporting repositories, we catalogued 471 EV‐related clinical trials to date, with indications for over 200 diseases. Diagnostics and companion diagnostics represented the bulk of EV‐related clinical trials with cancer being the most frequent application. EV‐related therapeutics trials mainly utilized mesenchymal stromal cell (MSC) EVs and were most frequently used for treatment of respiratory illnesses. Ultracentrifugation and RNA‐sequencing were the most common isolation and characterization techniques; however, methodology for each was not frequently reported in study records. Most of the reported characterization relied on bulk characterization of EV isolates, with only 11% utilizing EV subpopulations in their experimental design. While this may be connected to a lack of available techniques suitable for clinical implementation, it also highlights the opportunity for use of EV subpopulations to improve translational efforts. As academic research identifies more chemically distinct subpopulations and technologies for their enrichment, we forecast to more refined EV trials in the near future. This review emphasizes the need for meticulous methodological reporting and consideration of EV subpopulations to enhance the translational success of EV‐based interventions, pointing towards a paradigm shift in personalized medicine.

## 1 INTRODUCTION

Extracellular vesicles (EVs) have been implicated in countless physiological processes and diseases and are major emerging players in next‐generation diagnostic and therapeutic platforms. EVs are nanoparticles delineated by a lipid bilayer surrounding a hydrophilic lumen and are produced by cells through a variety of biogenesis pathways. The incorporation of functional molecules of interest (proteins (Zhang et al., 2017), lipids (Subra et al., 2010), nucleic acids (Fong et al., 2015), carbohydrates (Williams et al., 2019)), which can be adsorbed to the surface (Cvjetkovic et al., 2016; Wolf et al., 2022), embedded in the membrane (Giovanazzi et al., 2023; Rai et al., 2021), or contained in the EV lumen (Emelyanov et al., 2020; Ko et al., 2023), confers cellular signalling both near the cell of origin (Frühbeis et al., 2020; Microbiology, Society) and in distant tissues (Banks et al., 2020; Hoshino et al., 2015). While studies elucidating their diverse cargos and resulting functions is well underway in the context of basic cell biology and pathophysiology, EVs are also being applied in human clinical diagnostics and therapeutics.

EV based diagnostics largely focus on identifying the physicochemical signatures of EVs produced during disease initiation and development. Compared to conventional invasive tissue biopsy, often used to diagnose cancers, certain inflammatory disease (e.g., Celiac disease), and other conditions, tissue derived EVs are found in peripheral circulation and can be isolated through minimally invasive collection (e.g., blood, urine, saliva) to report on the disease of interest, rendering them an attractive target for liquid biopsy diagnostics (Irmer et al., 2023). Cancer associated EVs have been the most well studied in this regard, with EV proteins (Tian et al., 2021), nucleic acids (Su et al., 2021), chemical spectral signatures (Koster et al., 2021), and even concentration (Osti et al., 2019) changing during disease. Distinct EV signatures have also been identified in many other types of diseases including neurodegenerative (Cano et al., 2023), cardiovascular (Ji et al., 2016), and infectious diseases (Zipperle et al., 2023). EVs have been widely reported to have potential for initial diagnosis, as well as monitoring progression and response to therapy (Kassam et al., 2019; König et al., 2017) for either difficult to diagnose (e.g., Alzheimer's disease) or other ‘silent’ diseases (e.g., diabetes).

EV based therapeutics have sought to leverage the characteristics of EVs that aid in intercellular communication such as native functional effects from associated cargo and beneficial interactions with target cells. Mesenchymal stromal cell (MSC) derived EVs have been the most commonly explored in this context, due to their regenerative and protective properties in many conditions (Kim et al., 2021; Li et al., 2021; Liu et al., 2020). However, EVs from other human (e.g., dendritic cells (Pusic et al., 2014)) or non‐human (e.g., lemon juice (Raimondo et al., 2015), bacteria (Huang et al., 2014)) sources have also been considered as therapeutics or vaccines. Unmodified, native EVs are a promising alternative to cell therapies with less complex and longer‐term storage requirements and an inability to replicate due to their cell‐free nature, while retaining their desirable therapeutic benefits. Moreover, EVs can be further engineered and tailored for particular functions, such as loading of exogenous cargo or theranostic payloads (Cheng & Hill, 2022; Escudé Martinez de Castilla et al., 2021).

One factor that may either help or hinder use of EVs in the clinic is their inherent heterogeneity in both physical (size (Miroshnikova et al., 2023), density (Willms et al., 2016), lamellarity (Bobrie et al., 2012; Emelyanov et al., 2020; Zabeo et al., 2017)) and chemical (protein (Spitzberg et al., 2023), nucleic acid (Albanese et al., 2021), charge (Brown et al., 2020)) characteristics. While heterogeneity is often identified between EVs isolated from different cell types, this heterogeneity is also present within EVs from a single cell type (Bordanaba‐Florit et al., 2021; Kowal et al., 2016; Phillips et al., 2021). EV heterogeneity can also evolve in vivo as a result of changing disease status and metabolic stressors based on age (Eitan et al., 2017), sex (Enjeti et al., 2017), recent exercise (Frühbeis et al., 2015), and more. Alongside this inherent heterogeneity, exogenous factors can also lead to changes in EV physical characteristics and function such as isolation technique (Veerman et al., 2021), storage conditions (Gelibter et al., 2022), and route of administration (Kang et al., 2021; Wiklander et al., 2015). While the field is still identifying some of the factors that impact EV characteristics and function, there has been a great push for rigorous record keeping of EV related protocols to ensure that these factors are controlled and chosen purposefully (Théry et al., 2018; Van Deun et al., 2017; Welsh et al., 2024). While EV heterogeneity is a fast‐growing topic in pre‐clinical work, the impact of EV heterogeneity on clinical trials has gone largely unconsidered. While changes to EV cargo in disease state directly allow for their use in diagnostics, heterogeneity may also hinder their use. For example, it is unclear if certain disease‐related cargoes exist in specific subpopulations of EVs (i.e., from a specific cell type or even a subpopulation within EVs from a single cell type.) If this subpopulation were not identified, sensitivity of diagnostics may be lowered. In therapeutics, it is unclear if a subpopulation of EVs is at fault for a functional effect or if multiple subpopulations provide multiple functions. If a non‐active or deleterious subpopulation exists, this may lower the effectiveness of EV‐based therapeutics. Given that such a large range of factors can impact EV characteristics and functions, it is likely that these factors are greatly impacting downstream success in translational trials (Wakker et al., 2023).

To explore this premise and provide a perspective on clinical applications of EVs to date, we completed a systematic review to catalogue and analyse the landscape of all reported EV‐related clinical trials. By employing a systematic review methodology, we aim to offer a comprehensive overview of the trials, allowing us to identify the current applications and methodologies employed in the field and to discern emerging trends and gaps in the existing research, especially in the context of EV heterogeneity to shed light on future translational research.

## 2 SYSTEMATIC REVIEW

### 2.1 Methods

**FIGURE 1:**
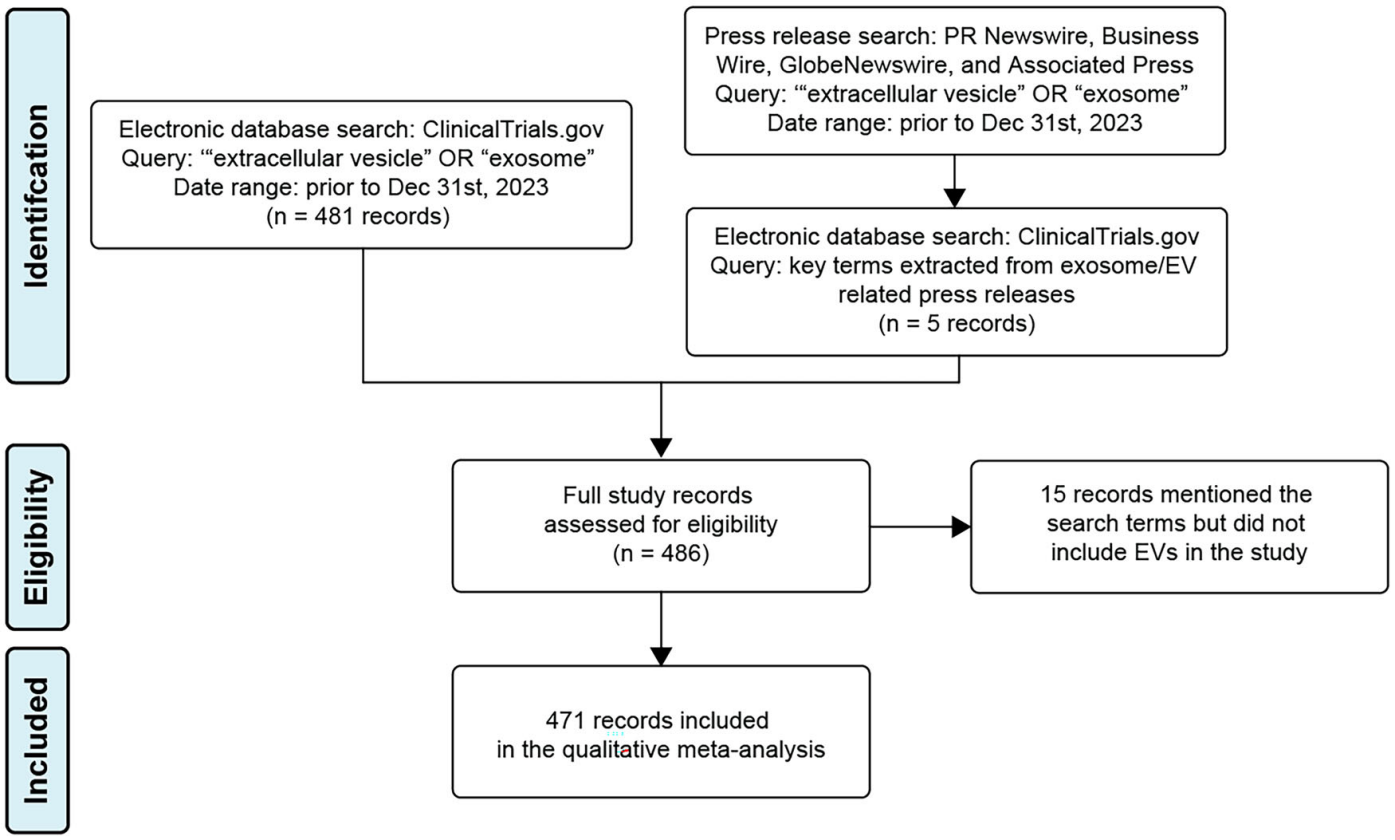
Flow diagram of the study inclusion and exclusion.

To perform this systematic review, we searched ClinicalTrials.gov with the query ‘extracellular vesicle’ OR ‘exosome’ and tabulated all entries that were returned. We included clinical trial records with a date first posted on or before 31 December 2023 (486 records, in Table S1). Expanded access protocols were also included. We noted that this query did not return several prominent EV‐related clinical trials known to our study team, since their associated record in ClinicalTrials.gov referred to the EVs with custom nomenclature. Therefore, we additionally queried press release databases (PR Newswire, Business Wire, GlobeNewswire, and Associated Press) utilizing the same search terms in the same period. Key terms gathered from press releases of interest were used to find associated records (if any) in ClinicalTrials.gov, resulting in identification of five additional study records (Table S2). Exclusion criteria were: (1) study records identifying EVs as a mechanism of action but not the main component (e.g., cell therapies or devices that remove EVs from circulation), and (2) study records which had no indication that EVs were involved in the study, despite naming them in the record (e.g., sponsored by an EV related company, or suspected misspelling (usually of exome)). In total, 15 study records were excluded by these criteria (Table S3), eight involving EVs as a mechanism of action and seven that did not use EVs. A flow diagram of this methodology is presented in Figure 1.

### 2.2 Limitations of the methodology

ClinicalTrials.gov does not require extensive reporting of methodologies within study records and, in trials that involve potential future patents or proprietary information, records may be written with the intention of being vague. Consequently, the degree to which many trials reported various EV‐related methodologies varied greatly between study records as well as the clarity with which certain techniques were assigned to EVs or other biospecimens. This means that this data only provides an estimate of trends in the field and likely underreports more specific details of these trials. A limited number of records included in our study have linked manuscripts or potentially non‐public protocols with more thorough reporting of various study protocols, methodologies, and outcomes, but these were generally not considered in our qualitative analysis presented below. Additionally, though ‘exosome’ and ‘extracellular vesicle’ are the most common terms in pre‐clinical and clinical research for EV‐studies, there may have been studies focused on additional subtypes of EVs (e.g., microvesicles, migrasomes, oncosomes, exomeres, ectosomes) not included in our analysis. An additional limitation is that this study only included trials registered at ClinicalTrials.gov. While this is the most used repository for clinical trials around the world, it is possible that there are clinical trials that would have fit our criteria on other clinical trial repositories such as the World Health Organization's International Clinical Trials Registry Platform or EU Clinical Trials Register since this repository is based in the United States. However, upon querying additional registry platforms, each had substantially fewer entries than ClinicalTrials.gov, with the World Health Organization's International Clinical Trials Registry Platform having the most at 177, suggesting that our search covered most EV‐related trials on registration platforms. Finally, some study records appear to be continuations of previous trials with only limited changes besides dates. While initially we intended to exclude some of these duplicates, it was difficult to determine a criteria for the degree of change that constituted a ‘new’ trial, so we elected to leave them for consistency. Though this may artificially increase the percentage of certain conditions or other characteristics, these study records were a minority of those included, and likely did not have a large impact. In addition, study records are updated continuously, with record versions included in these studies. Since study data was pulled beginning December 2022 up to February 2024, there is the possibility that some study data was updated within that time. Furthermore, the same data pulled later may result in some changes to data (e.g., study participants changing from an estimate to an actual number, or a study leg being added including EVs).

## 3 RESULTS

**FIGURE 2:**
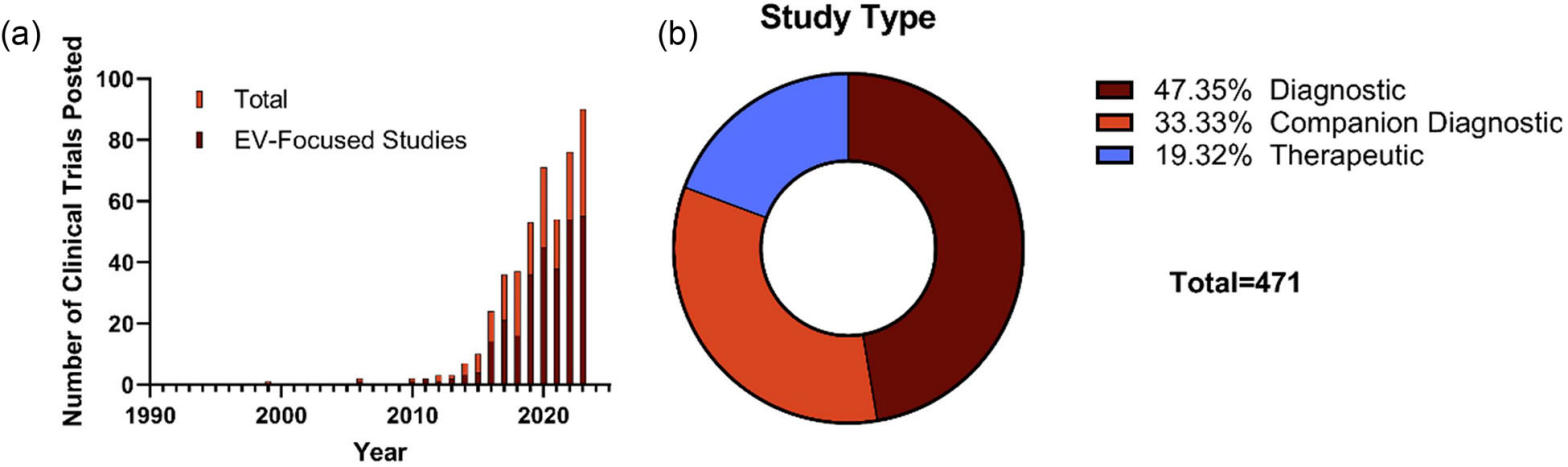
Total EV‐related clinical trial description. A timeline of study record posting dates (a) and study type (b) for EV‐related clinical trials identified via ClinicalTrials.gov shows an exponential rise in EV‐related clinical trials the majority of which are for diagnostics.

A total of 471 EV‐related clinical trials were included in our analysis (Figure 2). Seventy‐nine trials (17%) were industry sponsor initiated, while the remaining studies were investigator initiated. While we did not find any clinical trial specifically isolating exosomes (i.e., EVs specifically produced through intracellular fusion of multivesicular bodies (MVB) with the cell membrane), this term has historically been used to describe a heterogeneous isolate including exosomes and other EV types (Couch et al., 2021; Witwer & Théry, 2019). These other EV types can include ectosomes, oncosomes, migrasomes, apoptotic bodies, and others, each with unique biogenesis routes and/or chemical features, yet these subtypes are difficult to separate due to overlapping characteristics. Roughly two thirds of the trials here utilized the term ‘exosomes’ exclusively. In this review, we will use the broader term EVs to refer to the particles of interest throughout (Witwer & Théry, 2019).

The first EV‐related clinical trial (a diagnostic study) was posted in 1999 (Figure 2a); however, trials were only consistently posted beginning in 2010 with over half being posted after 1 January 2020. As clinical trials typically include a central goal in addition to several exploratory sub‐goals, our search criteria included studies in which EVs were not the main component being tested. To better understand the proportion of EV‐focused clinical trials, trial records in which EVs were a component of the title or were included within a primary outcome were tabulated, constituting 62.2% of all trials with this percentage remaining similar since 2010. While not a perfect metric due to the non‐standardized reporting, this gives a reasonable estimate for trials with a main focus on EVs.

For all study records, a median of 69 patients were enrolled, with a maximum of 23,000 patients in one study (Figure S1a). This median has not dramatically shifted since 2010, but the upper range does appear to have shifted upwards in recent years (Figure S1b). Clinical trials were most commonly located within the United States (27.6%), China (20.17%), or France (9.34%) (Figure S2). Only 15 of the included clinical trials here had been terminated (as of 28 July 2024). While this does not encompass all ‘failures’ of clinical trials since a clinical trial can be completed but not move on for a following phase or fail to be approved, it is a useful proxy that is reported on clinicaltrials.gov. Of all trials, only 15 had reported results on clinicaltrials.gov (as of 31 December 2023), five of which had been terminated.

Nearly half (47.4%) of EV‐related clinical trials examined EVs as diagnostics (defined as being used during diagnosis or for stage/sub‐type identification) with an additional 33.3% examining EVs as companion diagnostics (defined as being used alongside a therapeutic to track patient status, triage for a certain therapeutic arm, or monitor response to therapy) (Figure 2b). Therapeutics constituted the remaining 19.3% of EV‐related clinical trials. To better understand trends in each of these applications, we further analysed each record within each group of diagnostics, companion diagnostics, and therapeutics.

**FIGURE 3:**
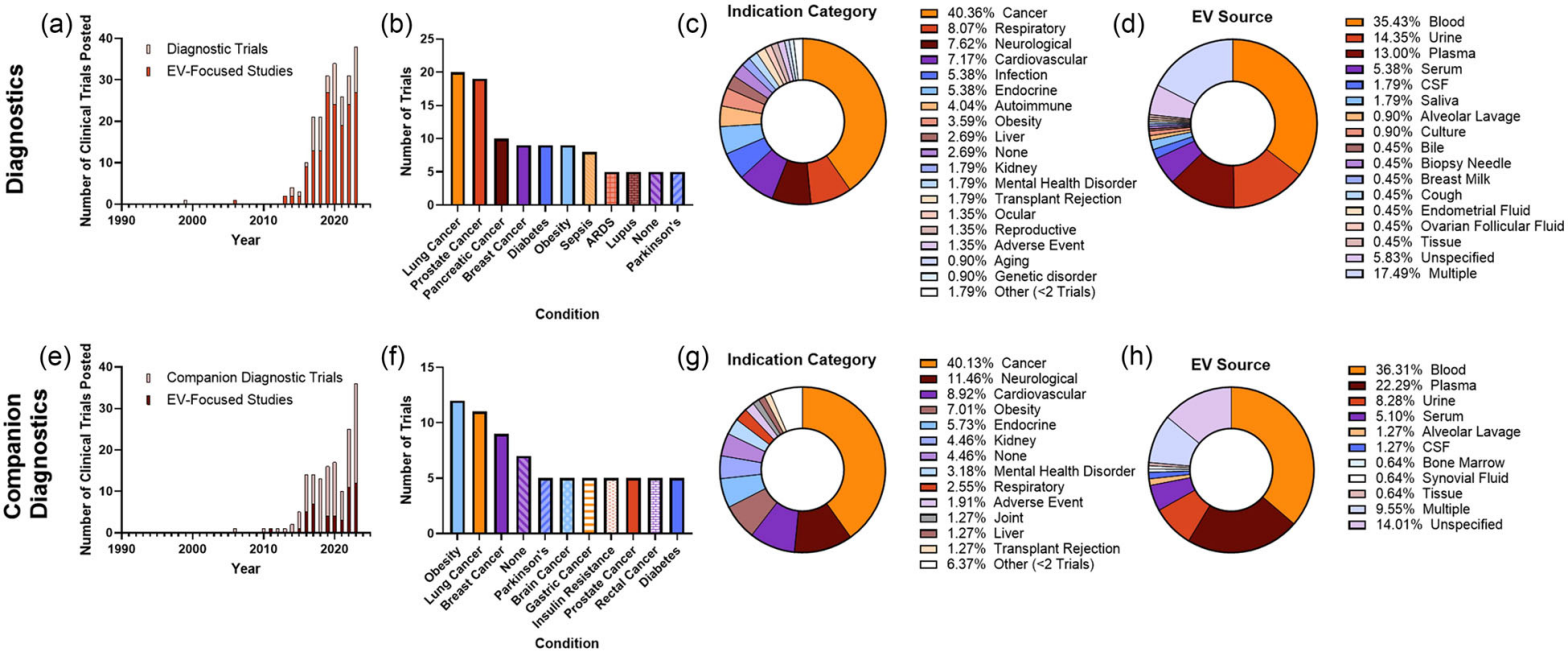
Summary of EV‐related diagnostic and companion diagnostic clinical trials. (a, e) Number of trials posted by year shows most diagnostic trials were posted in the late 2010s and early 2020s and the majority are EV focused. Most frequent conditions (≥5 trials) (b, f), general indication category (c, g) show that the majority of trials focus on various cancers. For both trial types, the majority of studies utilized blood or blood derivatives as an EV source (d, h).

The first EV‐related diagnostic trial was posted in 1999, with consistent trials starting in 2013 (Figure 3a). 73.1% of all EV‐related diagnostic trials were EV‐focused. Non‐EV focused trials typically included EVs in the secondary outcomes and were exploratory without a detailed description of their use. EV‐related diagnostic trials were applied to a diverse group of conditions, with a total of 120 individual conditions named. There were 11 conditions with at least five clinical trial study records, lung and prostate cancer being the most common listed with 20 and 19 trials respectively (Figure 3b). When conditions were loosely categorized, cancer and respiratory illnesses (e.g., COVID related illnesses, asthma) constituted roughly half of the registered diagnostic clinical trials (Figure 3c). A source for EVs was identified in 94.2% of trials and was mainly blood or blood derivatives for a total of 53% of trials (Figure 3d). 17.5% of trials used multiple biofluids, of which the top three were blood (25 trials), urine (12 trials), and plasma (11 trials).

The first EV‐related companion diagnostic trial was posted in 2006, with consistent trials being posted in 2010, like the trend seen for diagnostics (Figure 3e). Only 30.6% of companion diagnostic clinical trials were focused on EVs. The majority of non‐EV focused clinical trials had a primary outcome of safety or efficacy of a therapeutic, EVs being a secondary exploratory outcome to track patient response. The conditions studied in these trials had similar diversity to EV‐based diagnostics with 80 individual conditions identified (Figure 3f). Obesity was the most common condition being studied in 12 separate trials (Figure 3g). When categorized, companion diagnostics appeared to have similar applications to diagnostics the most common being cancer (40.1%), and the main notable changes being an increase in neurological conditions (11.5%) and a decrease in respiratory conditions (2.6%). A source for EVs was identified in 86.0% of trials and was largely identified as blood, serum, or plasma (when specified) for a total of 63.7% of trials. 9.6% of trials used multiple biofluids (Figure 3h) with the most common combined biofluids being blood (seven clinical trials), tissue (six clinical trials), and plasma (five clinical trials).

**FIGURE 4:**
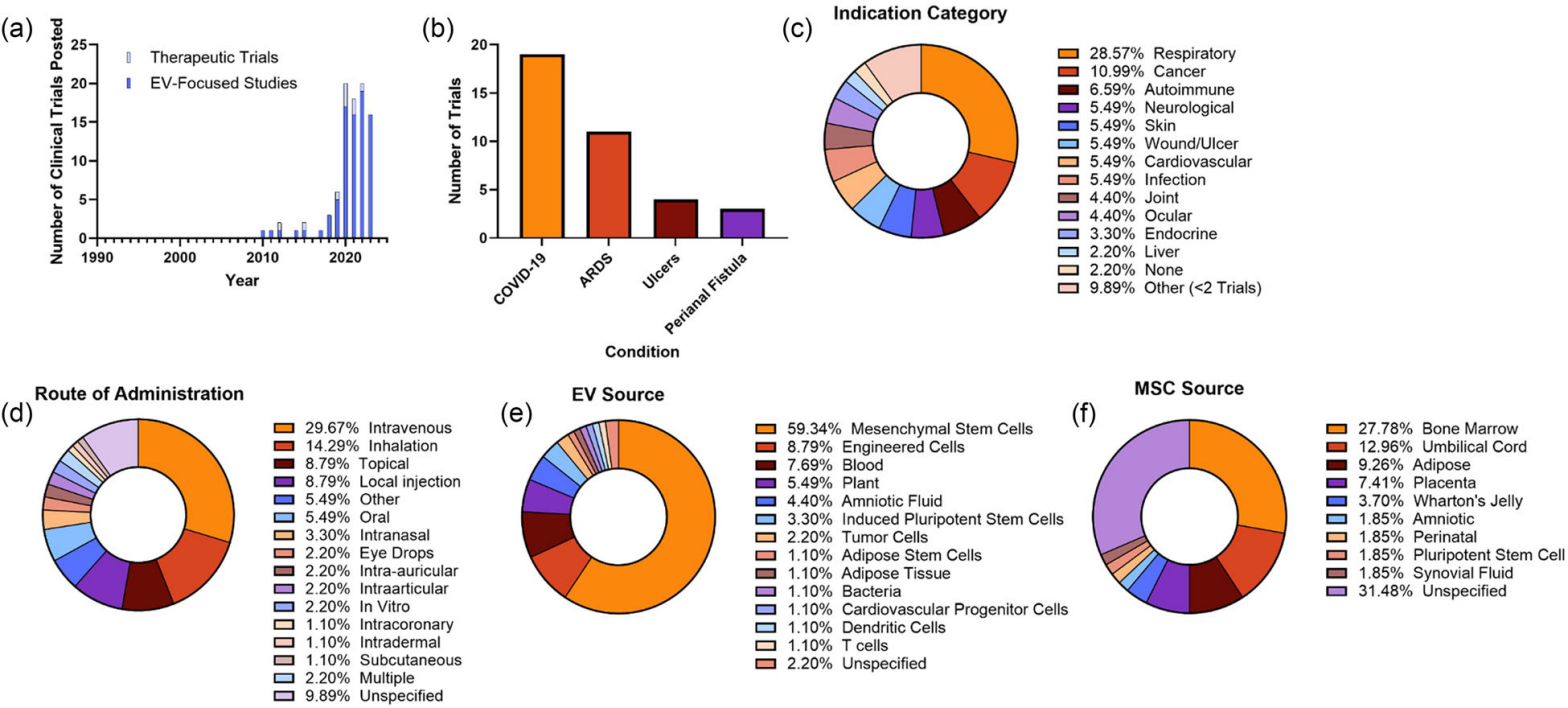
Summary of EV‐related therapeutic clinical trials. (a) All EV‐related therapeutic trials were posted since 2010, and the majority are focused on EVs. Most frequent condition (≥3 trials) (b), indication category (c), EV source, (d) MSC source (for trials utilizing MSC‐EVs), and route of administration were tabulated showing a large proportion of therapeutics intended for respiratory illnesses and largely being sources from MSCs.

The first EV‐related therapeutic trial was posted in 2010, with consistent trials beginning in 2017, a notably later timeline than identified for either diagnostic type (Figure 4a). 90.1% of EV‐related trials were EV focused (the highest of all study types), with the remaining 9.9% mainly utilizing cell biofluid products which contained, but were not specific to, EVs. Similar to diagnostics and companion diagnostics, there was a large diversity in the number of conditions being treated in EV‐related therapeutics trials, with 61 individual diseases. Only two conditions had more than five clinical trial study records, with the majority being for COVID‐19 (both acute and long) and acute respiratory distress syndrome (ARDS) (Figure 4b). Ulcers and perianal fistula each were indicated in at least three clinical trials. Most investigated respiratory illnesses (28.6%), and along with cancer (11.0%), and autoimmune conditions (6.6%), constituted nearly half of all therapeutic studies (Figure 4c). 90.1% of studies specified route of administration, with intravenous being the most common (29.7%), inhalation the second most common (14.3%), likely tied to the number of respiratory illnesses being treated, and topical being the third (8.8%) (Figure 4d). Most studies (59.3%) utilized EVs from MSCs (Figure 4e). This was followed by engineered cells (8.8%), blood (7.7%), whether autologous or allogeneic, and plants (5.5%). Of trials utilizing MSC‐EVs, MSCs were largely sourced from bone marrow (27.8%) and the umbilical cord (13.0%), though many study records did not specify (31.5%) (Figure 4f).

While information of dosing was often not thoroughly reported, we tabulated the number of study records including this information for preliminary analysis. Thirty‐two studies (35.2%) identified a dosing strategy, with 24 of those studies using number of particles for dosing and an additional four using the number of producer cells for dosing. Protein dosing was likely more common than quantified here (one study), as at least eight studies specified a dose in grams (µg or mg); however, since dosing by lipid is also a potential strategy (one study here), we considered these studies unspecified. In addition, 51 studies specified that patients would have multiple administrations of the therapeutic and 28 studies specified multiple doses would be tested (i.e., concentrations).

**FIGURE 5:**
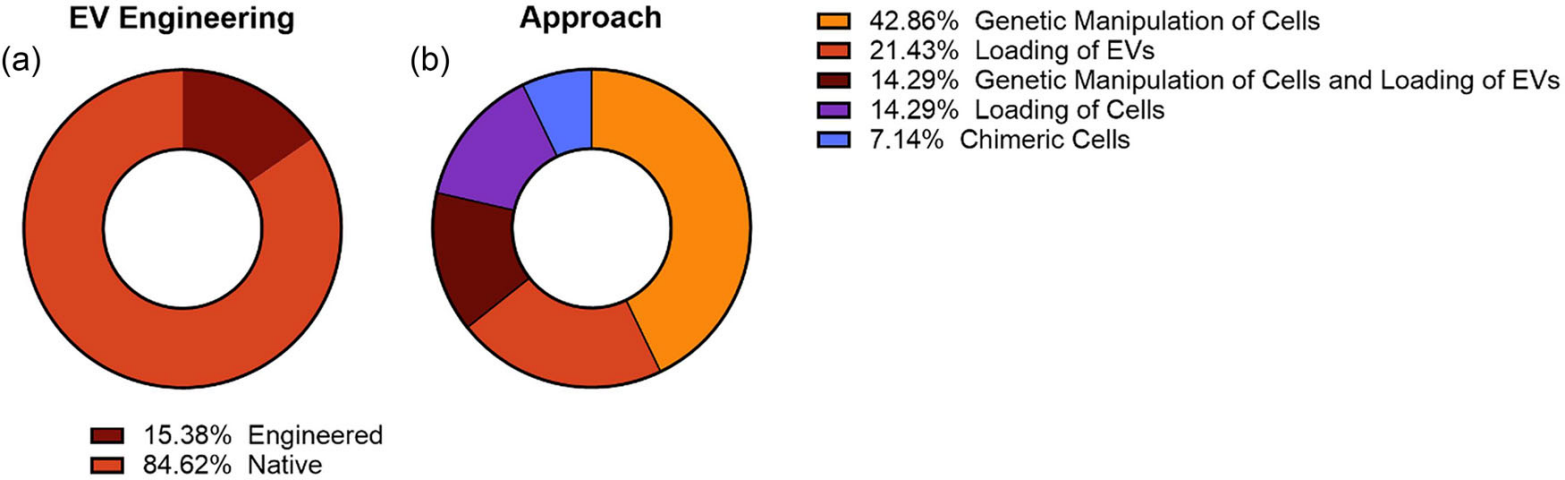
Summary of engineered EVs in EV‐related therapeutic trials. The proportion of trials utilizing engineered EVs (a) and the approach to EV engineering were tabulated (b).

Most EV‐based therapeutic trials utilized native EVs with only 14 (15.4%) utilizing engineered EVs (Figure 5a). The majority (eight) of these trials utilized gene editing of the parent cell line to manipulate EVs, two of which also added additional molecules to the isolated EVs (Figure 5b). Added molecules included proteins for targeting (e.g., PTGFRN, NCT04592484), proteins for therapeutic effect (e.g., super‐repressor IκBα, NCT05843799), nucleic acids for therapeutic effect (e.g., miRNA‐124, NCT03384433), and other chemicals for therapeutic effect (e.g., curcumin, NCT01294072) In addition, one study utilized EVs from chimeric cells produced from fusing patient tumour cells with antigen presenting cells (NCT05559177).

In addition to understanding the source and application, we identified trends in the procedures used to isolate and characterize EVs. Isolation techniques can greatly impact the presence and type of co‐isolate (Sharma et al., 2020), while choice of characterization techniques can majorly affect sensitivity to those readouts (Nieuwland et al., 2022). For example, some of the most common EV analysis platforms, that is, nanoparticle tracking analysis (NTA) or flow cytometry (FCM), are limited to detecting EVs larger than 70–80 nm, and therefore are largely insensitive the majority of EVs, which are 40–80 nm in diameter (van der Pol et al., 2022).

**FIGURE 6:**
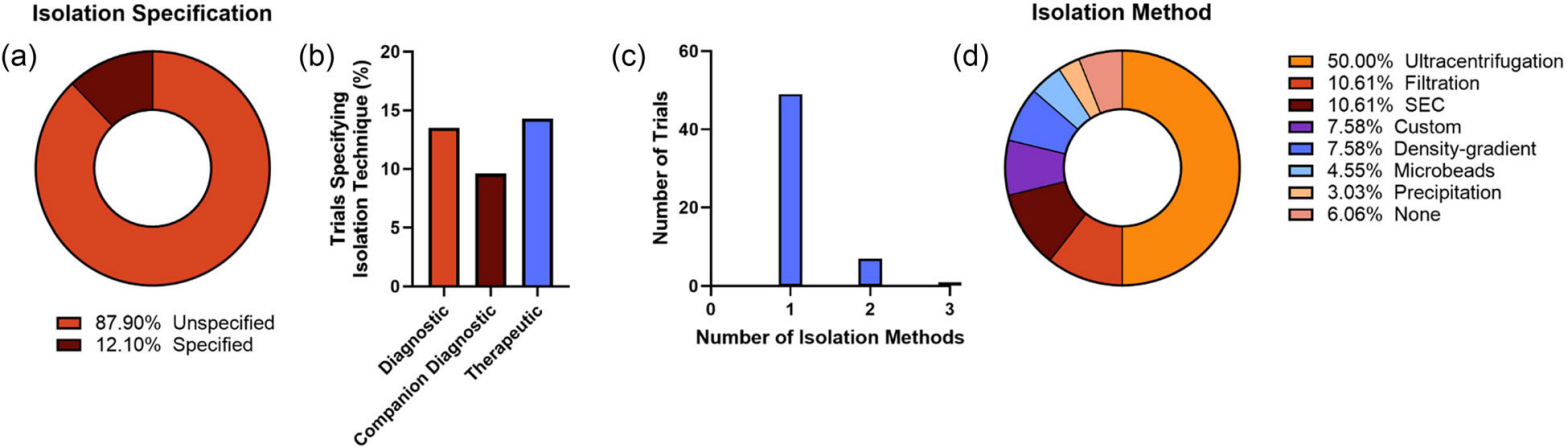
Summary of EV isolation methods in EV‐related clinical trials. Total study records identifying isolation method (a), and the frequency of specification by trial type (b) were quantified. The total number of isolation methods used per study were also quantified (c). Isolation method was determined as a percentage of all isolation methods identified (not as percentage of trials) (d).

Only 12.1% of studies reported the isolation methodology in the study record (Figure 6a). Therapeutics trials were more likely to report isolation methodology as compared to either type of diagnostic trials (Figure 6b). Of the trials that identified an isolation method, the majority (86.0%) utilized only a single method, ultracentrifugation being the most popular (Figure 6c,d). This was followed by filtration and size exclusion chromatography (SEC).

**FIGURE 7:**
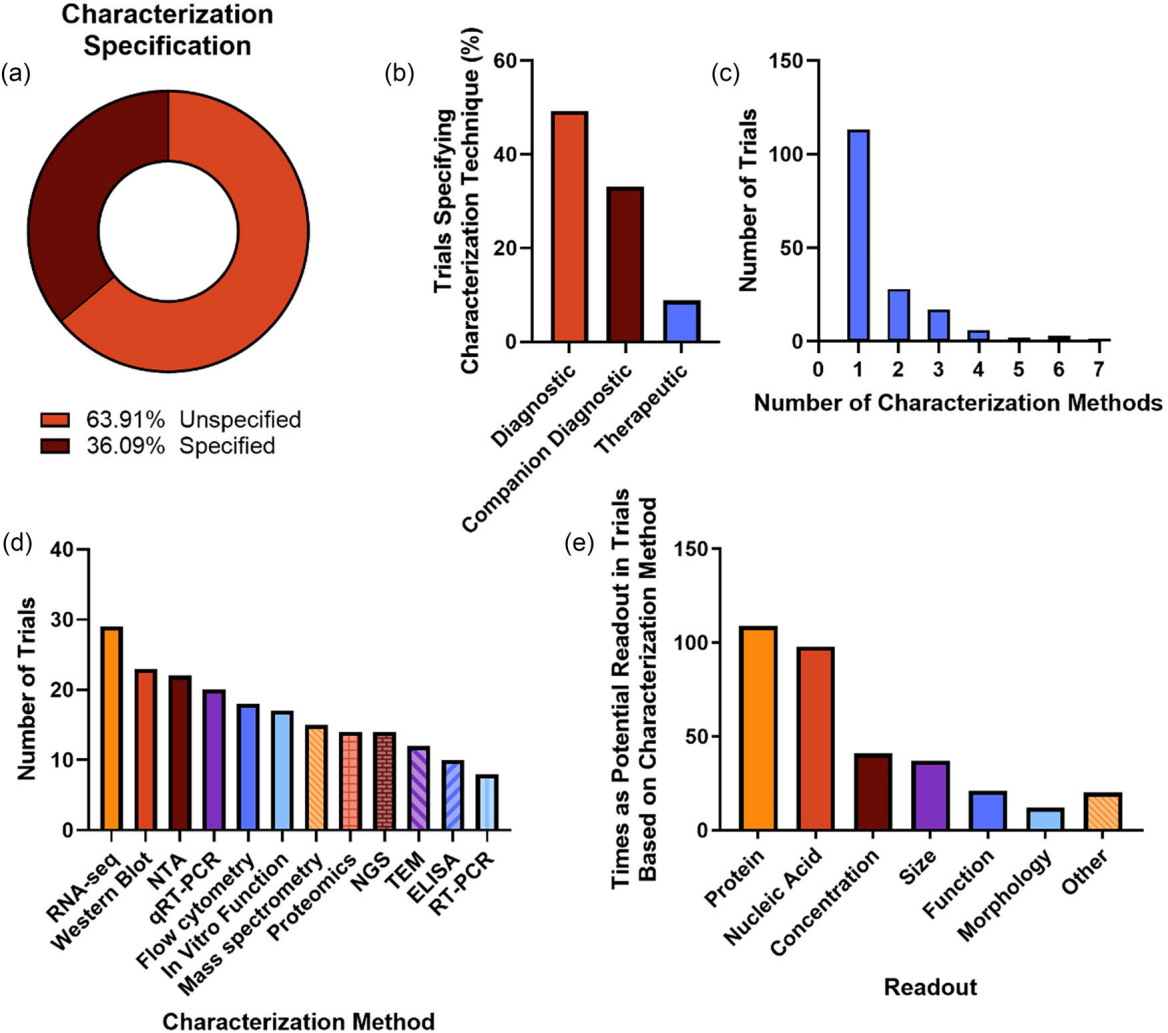
Summary of EV characterization methods in EV‐related clinical trials. Study records identifying any characterization methods (a) and total number of characterization techniques identified (c) were tabulated showing that characterization method was overwhelmingly unreported. Most commonly identified characterization techniques were identified across all trial (≥8 trials) and shown in (a). In addition, characterization techniques were categorized to determine the readout and tabulated to show trends in molecules and characteristics of interest (e).

Surprisingly, only 36.1% of studies identified at least one characterization method (Figure 7a) with diagnostic trials being the most likely to identify any method (Figure 7b). Reporting the use of a single characterization method was most common (66.5%), though up to seven were identified in a single study (Figure 7c). RNA‐sequencing (RNA‐seq) was the most common characterization method, followed by Western blot, nanoparticle tracking analysis (NTA), qRT‐PCR, and flow cytometry (Figure 7d). To better generalize characterization, we tabulated the common potential readouts for the characterization techniques identified in study records to estimate trends in the characteristics being studied in these clinical trials. Overall, the most common potential readout was protein expression, followed by nucleic acids, concentration, and size (Figure 7e). This analysis is limited in that many records were difficult to interpret and identify if a specific technique was being used to analyse EVs or a different biospecimen, as many studies included multiple biospecimen types. Only techniques that appeared to be specifically applied to EVs were included. While this is not an exhaustive list of potential readouts (e.g., some studies indicating identifying lipid types), these characteristics convey the majority of readouts that were identified in these trials.

**FIGURE 8:**
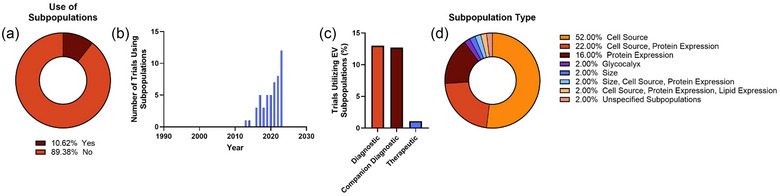
Summary of EV subpopulation uses in clinical trials. Fractions of study records identifying use of or attention to an EV subpopulation (a) and the number of each of these trials per year (b). These trials were broken down to show the frequency of subpopulation consideration in each trial type (c) and by subpopulation parameter (d) with a majority focusing on cell source subpopulations.

Finally, we assessed the number of studies that utilized EV subpopulations as a part of their research design. Only studies in which there was an indication that a subpopulation would be selected for or separated from the bulk were considered (e.g., percentage of EVs expressing CD9 by flow cytometry) as compared to identifying a bulk property of an EV isolate (e.g., high expression of CD9 by ELISA). Only 10.6% of studies indicated use of EV subpopulations; however, consideration and use of these subpopulations appears to be delayed compared to onset of all clinical trials and may continue to increase in the coming years (Figure 8a,b). Diagnostics and companion diagnostics were much more likely to utilize subpopulations than therapeutics (comparing therapeutic effect of exosomes and microvesicles, NCT02138331) (Figure 8c). Identifying EV subpopulations from a specific source was the most common indication of subpopulation use, followed by identification of a specific protein (Figure 8d). Many of these trials indicated that identifying concentrations of EVs from various cell sources was the intended outcome, often with a connection to the associated disease. For example, in study record NCT05451342, the levels of peripheral blood mononuclear cells and alveolar macrophage derived EVs in blood and alveolar lavage between patients with and without ARDS. Some studies took this a step further, isolating specific cells by a given characteristic and further probing for biomolecules, such as study record NCT05624203, separating EVs from endothelial progenitor cells and further probing this subpopulation for miR‐140‐3p expression in patients being treated for myocardial reperfusion injury.

## 4 DISCUSSION

While EVs have been studied in earnest in some capacity since the ∼1980s, the exploration of EVs as clinical therapeutics and diagnostics remains in its infancy. Since the first EV‐related clinical trial in 1999, we identified 471 total clinical trials mentioning either ‘extracellular vesicles’ or ‘exosomes’. The dramatic increase in clinical trials in recent years follows the progressive identification that EV production is affected in disease in the 1980s and 90s and later that EVs showed functional effects in vitro in 1996 and in vivo in 2007 EVs in 2007 (Couch et al., 2021).

Publication of study records has generally followed an exponential trend with the exception of recent years. However, this may be contributed to the decrease in clinical trials and revenue during the COVID‐19 pandemic in many fields (Bakouny et al., 2022; Qiu et al., 2021) (also seen in the reduced number of new EV clinical trials in 2020 compared to projections), these data suggest that the EV field will continue to exponentially grow.

Similar to prior reports, the majority of clinical trials have been registered for diagnostics or companion diagnostics (Ciferri et al., 2021). Frequently, these trials utilized biofluids already used for gold‐standard diagnostics, blood being the most common biofluid source of EVs. Cancer was the most common target for these diagnostics representing over one third of all diagnostic or companion diagnostic trials. This trend may be due to the ease of growing cancer cells in culture for pre‐clinical research and identification of diagnostic markers, while many other diseases cannot be as readily modelled in vitro. In addition, a liquid biopsy approach is particularly useful for cancer since many cancers are diagnosed via invasive tissue biopsy. Though trends varied slightly between diagnostics and companion diagnostics, EVs were largely applied across a small range of similar indications. Clinical trials for EV‐based therapeutics, in contrast, have mainly been explored for respiratory diseases. This was influenced by the COVID‐19 pandemic, with a disproportionate percentage of clinical trials for this application in the last 4 years (Sanz‐Ros et al., 2023). COVID‐19 and ARDS were the primary conditions treated in EV‐related clinical trials. EV therapeutics continue to be mainly sourced from stem or progenitor cells. These trends in applications for diagnostics and therapeutics appear to match those identified by others (Duong et al., 2023).

At least 204 unique conditions were represented in the 471 analysed clinical trials, even without considering many subcategories of specific illness (e.g., non‐small vs. small cell lung cancer). While this may be attributed to the youth of the field as it finds potential niches, this wide variety of applications can, in part, be tied to the heterogeneity of EVs as well as their broad potential to influence cells and tissues in many contexts. In diagnostics, the ability to detect the signatures of disease‐related EVs is directly related to this differential expression during disease‐state (Cuomo‐Haymour et al., 2022; Yoshioka et al., 2014). For example, a CD9^+^/CD147^+^ EV population was shown to be secreted into serum in colorectal patients and could be used for diagnosis or monitoring post‐surgery (Yoshioka et al., 2014). In therapeutics, the vast number of functions that often are specific to an EV source, aid in their application for many indications. For example, even different sources of MSCs for EV production (e.g., EVs produced from menstrual‐vs. bone marrow‐derived MSCs) appear to be best suited for treatment of different diseases when directly compared, such as for increasing neurite outgrowth (Lopez‐Verrilli et al., 2016) or for improving recovery post‐myocardial infarction (Xu et al., 2020). These studies show that EVs even from similar sources have different functions, likely owing to their compositional heterogeneity.

Yet only 10.6% of trials utilized EV subpopulations in their methods. In general, these studies employed identification of either a specific cell source for the EVs examined (though without specification for how this would be carried out) or a specific protein marker distributed across EVs. One example of consideration of subpopulations is study record NCT04578223, utilizing EVs as a companion diagnostic for response of pulmonary arterial hypertension to various drugs. This trial describes use of flow cytometry to quantify proportions of EVs produced from various cells by specific proteins to quantify platelet activation, suggesting that key subpopulations may differ during pathogenesis. While single‐particle flow cytometry was the most common method associated with identifying EV subpopulations, there were other techniques that were also used. For example, a study record for a stroke diagnostic, NCT05370105, utilizes a functionalized surface plasmon resonance imaging (SPRi) platform to capture EVs by various cell source markers (e.g., IB4 for microglia) and then probe for various markers of disease state (e.g., TSPO for microglial activation). Capture by specific proteins has been shown to impact sensitivity to differential mRNA expression in EVs (Fortunato et al., 2022). Intraluminal miRNA has been shown to be enriched in a small subpopulation of EVs, suggesting that functions may be resulting from only a small percentage of a total EV isolate (Albanese et al., 2021; Ko et al., 2023). These studies exemplify that the heterogeneity of EV isolates can impact sensitivity and function in previously unexpected ways, but also that this heterogeneity may be leveraged to improve clinical performance. As lack of efficacy is the main contributor to clinical trial failure (Sun et al., 2022), optimizing these subpopulations may be essential for success of EV translation to the clinic.

Interestingly, two single‐particle techniques, NTA and flow cytometry, were in the top five characterization methods identified in these study records. While single‐particle techniques can be used to describe bulk characteristics of EV isolates, they provide the opportunity to describe EV subpopulations. Both fluorescence NTA and single‐particle flow cytometry are able to identify subpopulations, albeit with different sensitivity, and could provide opportunities to better utilize these subpopulations (Welsh et al., 2023). However, even considering these two single‐particle techniques, most of the identified techniques are limited to bulk analysis (i.e., Western blot, RNA‐seq). Though these are useful, they are unable to characterize heterogeneity like recent single‐EV techniques such as super resolution microscopy or label‐free plasmonic sensing (Hilton & White, 2021). Increasing application of these single particle techniques and associated methods in clinical trial design could be a viable option for examining or utilizing subpopulations in the clinic. Admittedly, while not all these techniques are clinically adoptable due to low‐throughput, information gleaned from these techniques could better inform design of experiments to enhance efficacy of future diagnostics and therapeutics.

In addition, a surprising finding was that only 17 studies used in vitro or in vivo assays for functional assessment of their products. While in some cases this was due to the exploratory nature of these trials determining connection of disease with function of EVs, these assays could also be used to test potency of the product. For example, NCT05774509 specifies three separate tests to validate the activity of EV‐enriched secretomes. In addition, NCT04652531, though not specifying a specific in vitro test, mentions use of a potency assay prior to decision to administer the product. Defining potency assays for EVs remains complex, but will be an important step for their effective use in therapeutics (Gimona et al., 2021).

A noticeable trend was an overall lack of explicitly defined methodology. Only 12.1% of trials defined an isolation technique and only 36.1% defined any specific EV characterization techniques. It should be noted that this study was limited to examining the information within the study records submitted to ClinicalTrials.gov, and parts of the study design may have remained confidential or defined elsewhere. It is likely that many of these study records remain vague to avoid disclosure of potentially patentable or proprietary information. However, considering that nearly every study record described an EV source and, for therapeutic trials, a route of administration, a misunderstanding of the importance of these parameters likely also contributed. This lack of specification is in stark contrast to the research field where there has been a large effort to increase reporting of many of these parameters, as evidenced by MISEV reports (Théry et al., 2018; Welsh et al., 2024). Much of this push has been due to the heterogeneity introduced by some of these methods. Isolation method has massive impact on particles that co‐isolate with EVs (Sharma et al., 2020), but can also impact their functional capacity (Lang et al., 2022; Saludas et al., 2022). While characterization techniques do not alter the isolate, their inherent limitations and biases can impact the appropriate interpretation of their results. Multiple reviews have also found this lack of reporting to be an area of weakness for EV clinical trials (Duong et al., 2023) and list the parameters suggested to be defined and reported (Lener et al., 2015). While adopting new methods or techniques requires time, better definition and reporting of these parameters could be a simple and impactful change to improve EV based clinical trials. Study record NCT03811600 provides an example of a thorough description of its methodology to utilize flow cytometry to examine the role of PD1/PDL1 expressing EVs in sleep apnoea, identifying isolation method, characterization technique, and intended markers. Inclusion of this information in study records will be helpful for researchers examining trends in the field, but also to ensure that these factors are well‐defined and chosen intentionally for a specific need. Another interesting emerging trend is in the application of non‐human EVs, specifically plant derived EVs. Plant nanoparticles (NPs) from species such as lemons (Raimondo et al., 2015), garlic (Song et al., 2020), ginger (Li et al., 2018; Zhang et al., 2016), ginseng (Cao et al., 2019), grape (Ju et al., 2013), grapefruit (Savcı et al., 2021; Zhuang et al., 2016), and many others have been reported to exhibit therapeutic effects in many contexts, including cancer (Cao et al., 2019; Raimondo et al., 2015), immunomodulation (Cao et al., 2019; Ju et al., 2013; Song et al., 2020), wound healing (Savcı et al., 2021), and the delivery of compounds across the blood brain barrier (Li et al., 2018; Zhuang et al., 2016). Due to the harsh preparation and isolation methods used in these studies (such as blending or juicing), most samples used in plant vesicle research are considered NPs and not purely EVs, thus are contaminated with intracellular vesicles, soluble proteins, and plant phytochemicals, all of which contribute to the observed therapeutic effects (Pinedo et al., 2021). We found 5 study records involving plant EVs, in cancer (2), endocrine disorders (2), and autoimmune disorder (1). All five trials involve plant NPs isolated from fruits and were delivered orally to patients. Issues of rigor, clarity and nomenclature inconsistencies present in the academic plant EV research are also present in these clinical trials. In addition to the study characteristics, we examine here, plant‐derived EV studies would also benefit from including information on purity and source (Pinedo et al., 2021).

Several factors limit the interpretation of the records examined in this study. Though the identification of these clinical trials was unbiased, the information provided in clinicaltrials.gov only pertains to registered clinical trials and is not exhaustive and likely represents a limited view of the study. This may have led to fewer methods being reported or definitions being stated than were truly used throughout the trial, so it is important to interpret this as an estimation of trends in clinical trials. While some study records indicated published work, many of the cited work appeared tangential to the clinical trial goals making it difficult to integrate. Clinical trials can also be amended over time, as evidenced by the version histories in many study records. Since data was pulled at multiple points from December 2022–February 2024, there may have been minor changes to some study records that could shift these results. While we do not expect this to change any conclusions, it means that the same analysis later may be necessary to see shifts in these trials. Another important limitation is that search terms used in this study were not extensive, including only ‘extracellular vesicles’ and ‘exosomes’. Though those terms are the most used for small EVs, they are far from exhaustive. Oncosomes, migrasomes, ectosomes and many others have been identified as sub‐categories of EVs and could also be examined for their use in clinical trials. Finally, clinicaltrials.gov does not have strict reporting requirements and many of the study records are vague and lack information that is likely included within procedures. Therefore, this analysis is meant to act as an approximation of the current field of EV clinical trials to help direct future work.

Interests in clinical applications of EVs are rapidly growing, with exponential increases in clinical trials in the last two decades. Here, we analysed study records of EV‐related clinical trials to help identify trends in the field and areas for improvement. Most EV‐related clinical trials focused on diagnostic and companion diagnostic applications, with a smaller proportion focused on therapeutics. However, for each trial type there are a wide variety of EV sources and applications, suggesting that EV heterogeneity contributes to their diverse use. EV characterization and isolation technique were overall poorly reported and similarly showed a wide diversity.

This review underscores the burgeoning interest and potential of EVs in clinical applications. Despite the promise shown by EVs, our analysis reveals critical gaps in methodological reporting and a lack of focus on EV subpopulations, which could significantly enhance the translational success of EV‐based interventions. To propel the field forward, it is imperative to adopt rigorous and standardized methodologies, leverage the unique properties of EV subpopulations, and ensure thorough functional validation of EV products. These advancements will pave the way for more effective and personalized EV‐based diagnostics and therapeutics, driving a paradigm shift in personalized medicine.

**Key future milestones.**

To advance the field of EV‐related clinical trials, it is crucial to address key areas:

- **Improved methodological reporting and classification**: There is a need for meticulous and standardized reporting of isolation and characterization methodologies. Improved transparency in these methods will facilitate reproducibility, comparison across studies, and better understanding of the impact of different techniques on EV properties and functions.
- **Incorporation of EV subpopulations**: Employing advanced techniques to identify and isolate specific EV subpopulations may enhance the sensitivity and efficacy/potency of clinical applications. Clarifying the classification of EVs, particularly distinguishing between EV types, is necessary to ensure accurate interpretation of clinical trial results and to maintain rigor in the field.
- **Adoption of single‐particle resolved techniques**: Increasing the use of single‐particle techniques could provide detailed insights into EV heterogeneity. These techniques should be integrated into clinical trial designs to better understand and utilize EV subpopulations
- **Validation studies**: Rigorous validation studies should be reported, focusing on standardizing methodologies, ensuring reproducibility, validating functionality and potency, confirming clinical relevance, and assessing long‐term stability and safety. Incorporating in vitro and in vivo functional assays to validate the activity and potency of EV products is essential. Defining and standardizing potency assays will help in assessing the therapeutic potential and ensuring the quality of EV preparations.

## AUTHOR CONTRIBUTIONS

**Rachel Mizenko**: Conceptualization (supporting); Formal analysis (lead); Methodology (supporting); Visualization (lead); Writing–original draft (lead); Review and editing (lead). **Madison Feaver**: Conceptualization (lead); Formal analysis (lead); Methodology (lead); Visualization (supporting); Writing–original draft (supporting). **Batuhan Bozkurt**: Formal analysis (supporting); Writing–original draft (supporting); Review and editing (supporting). **Neona Lowe**: Formal analysis (supporting); Review and editing (supporting). **Bryan Nguyen**: Formal analysis (supporting); Review and editing (supporting). **Kuan‐Wei Huang**: Formal analysis (supporting); Review and editing (supporting). **Aijun Wang**: Review and editing (supporting). **Randy Carney**: Conceptualization (lead); Methodology (lead); Writing–original draft (supporting); Review and editing (lead).

## CONFLICT OF INTEREST STATEMENT

The authors declare no conflicts of interest.

## Supporting information



Supporting information

Supporting information
