# Supplementary material for: A critical systematic review of extracellular vesicle clinical trials
Source: J Extracell Vesicles. 2024 Sep 27;13(10):e12510. doi: 10.1002/jev2.12510 (PMC11428870; doi:10.1002/jev2.12510)
Supplement: Supplementary file 2 — Supporting information [file JEV2-13-e12510-s002.docx]

**Supplementary Information for**

**A critical systematic review of extracellular vesicle clinical trials**

Rachel R. Mizenko^1,†^, Madison Feaver^1,†^, Batuhan T. Bozkurt^1^, Neona Lowe^1^, Bryan Nguyen^1^, Kuan-Wei Huang^1^, Aijun Wang^1,2^, Randy P. Carney^1,^*

^1^Department of Biomedical Engineering, University of California, Davis, California, USA

^2^Department of Surgery, University of California, Davis, California, USA

^†^These authors contributed equally

**Supplemental table 1: Study records included for analysis**

*Available as an .xlsx file uploaded alongside this study*

**Supplemental table 2: Added study records**

**
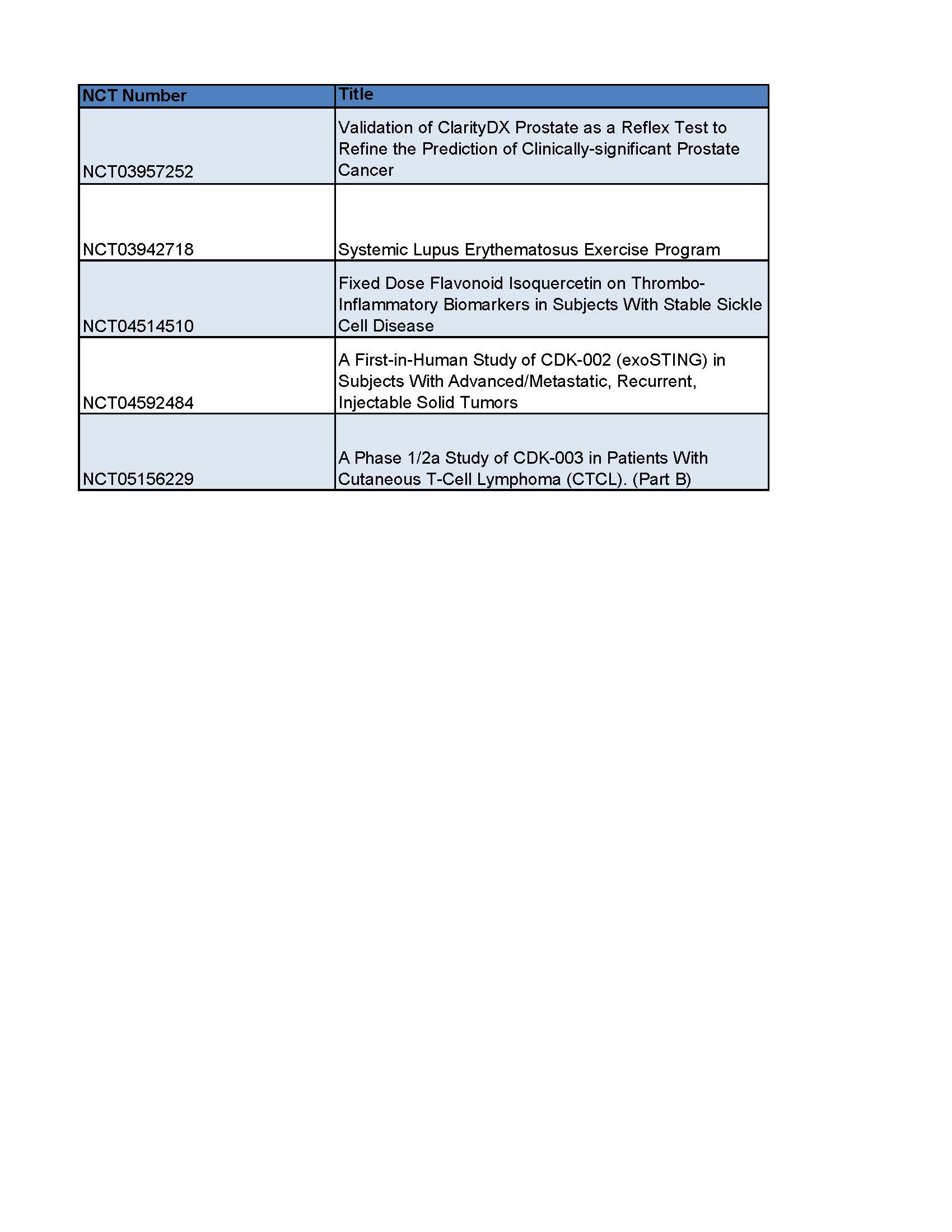
**

**Supplemental Table 3: Excluded Entries**

**
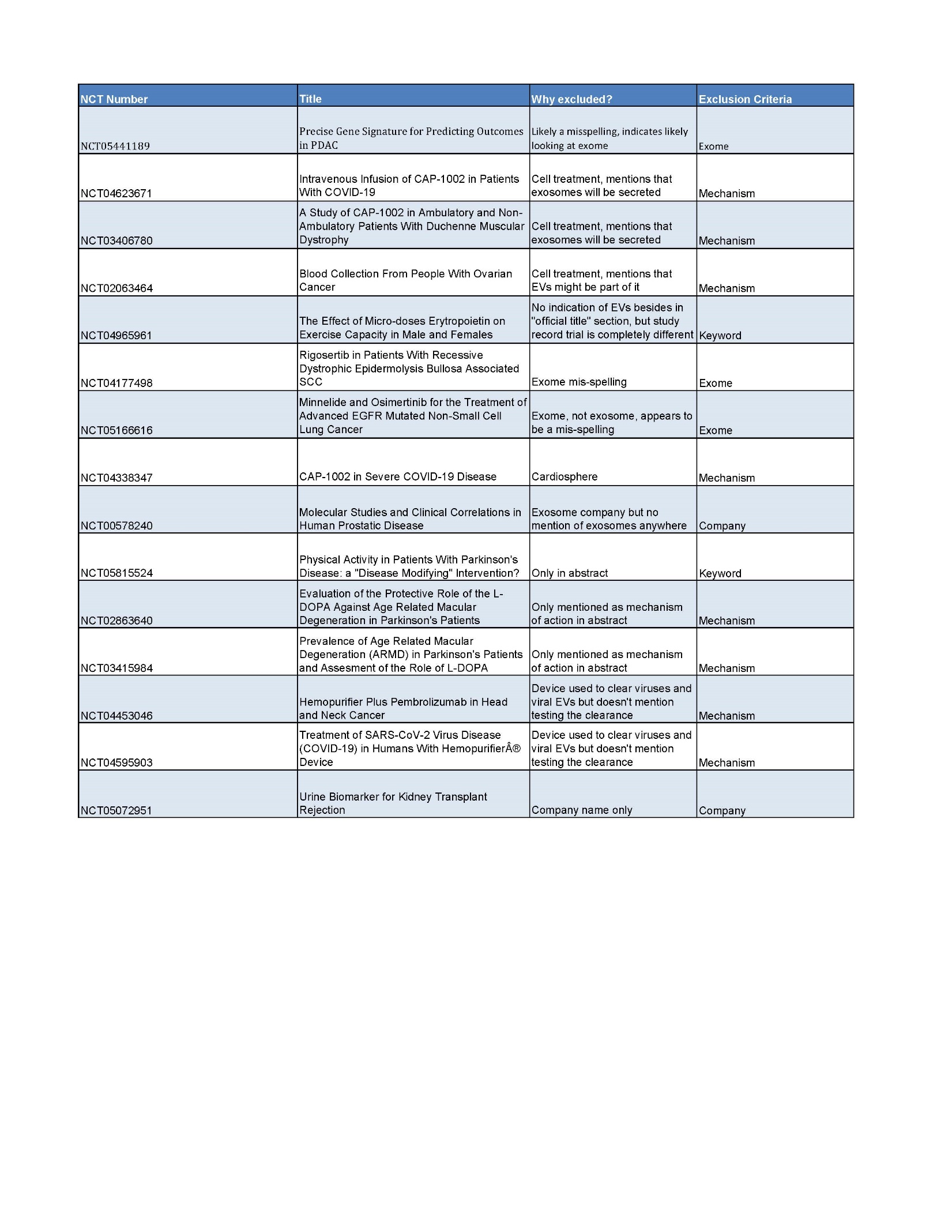
**

**
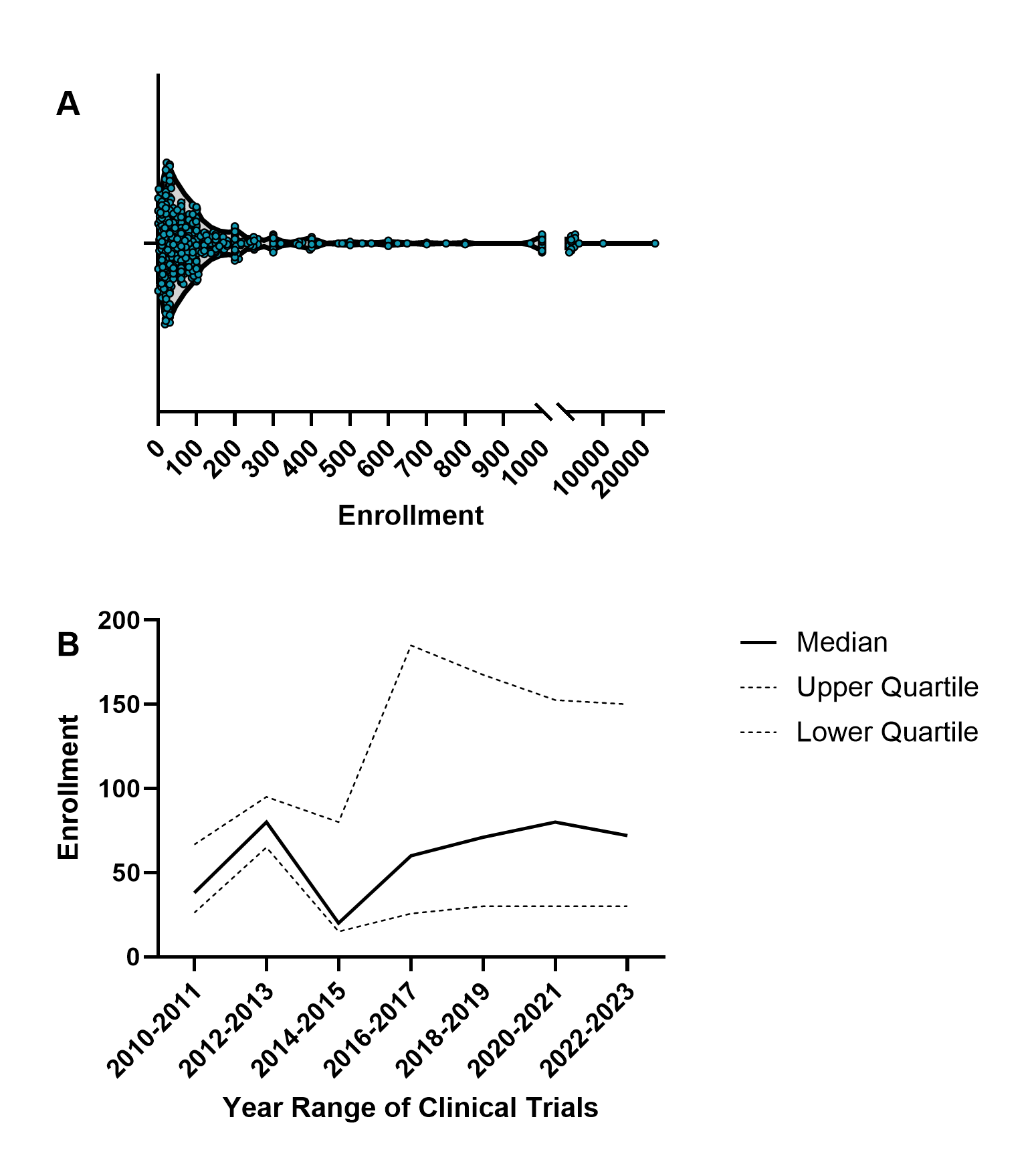
**

**Supplemental Figure 1: Patient enrollment in clinical trials. (A)** For all study records included in this analysis, the median number of patients enrolled was 69. Enrollment was also quantified over time **(B)** showing that though the median has not changed dramatically, the upper quartile has shifted upwards since 2015. If actual enrollment was not provided, estimated enrollment was used.

**
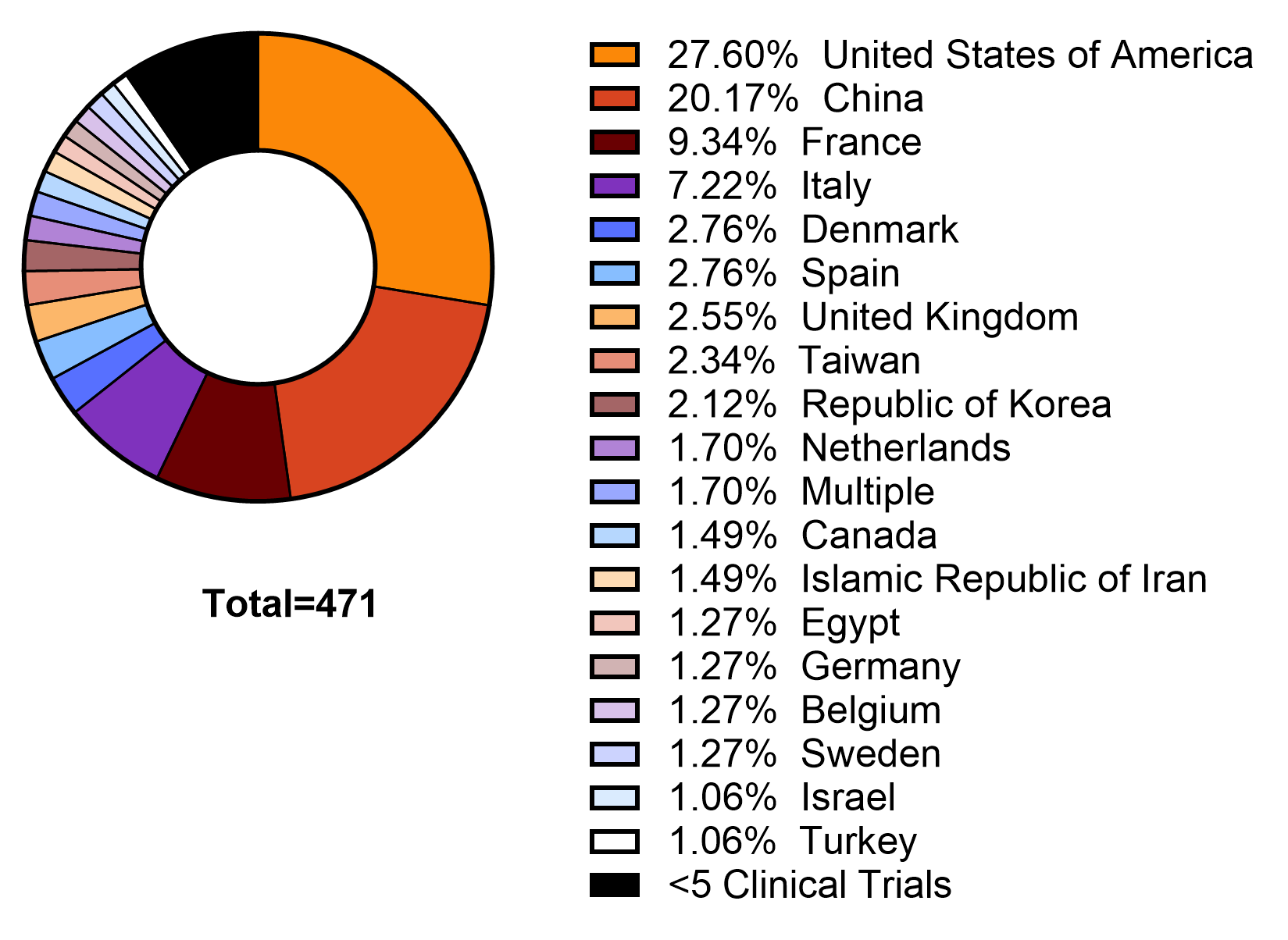
**

**Supplemental Figure 2: Locations of clinical trials.** The country conducting each clinical trial was tabulated and all countries with at least 5 reported trials are shown here.
